# Severe Multiple Sclerosis Relapse After COVID-19 Vaccination: A Case Report

**DOI:** 10.3389/fneur.2021.721502

**Published:** 2021-08-10

**Authors:** Giorgia T. Maniscalco, Valentino Manzo, Maria E. Di Battista, Simona Salvatore, Ornella Moreggia, Cristina Scavone, Annalisa Capuano

**Affiliations:** ^1^Multiple Sclerosis Center “A. Cardarelli” Hospital, Naples, Italy; ^2^Neurological Clinic and Stroke Unit “A. Cardarelli” Hospital, Naples, Italy; ^3^Department of Experimental Medicine, University of Campania “Luigi Vanvitelli”, Naples, Italy; ^4^Regional Center of Pharmacovigilance and Pharmacoepidemiology of Campania Region, Naples, Italy

**Keywords:** COVID-19 vaccine, multiple sclerosis, acute relapse, cladribine, case report

## Abstract

We describe a case of acute relapse in a woman with Multiple Sclerosis (MS) shortly after the mRNA COVID-19 vaccination. The patient received a diagnosis of MS in November 2016 at the MS Centre of the A. Cardarelli Hospital (South of Italy). Since that moment, her clinical conditions and pharmacological therapies have been managed at this MS centre where, according to national recommendations, in April 2021, the patient received the BNT162b2 vaccine. Almost 48 h after receiving the vaccine, the patient developed paraesthesia and weakness in her left arm and limbs. The neurological examination revealed walking difficulties while the MRI showed three new voluminous enhancing lesions. After having received methylprednisolone iv for 5 days, the patient's neurological symptoms fully recovered. Along with the implementation of COVID-19 vaccination programmes among vulnerable population, further studies are needed in order to improve our knowledge on the benefit/risk ratio of COVID-19 vaccines.

## Introduction

Following the approval of COVID-19 vaccines across EU countries, vaccination programmes have been started in order to identify vulnerable people at highest risk from serious illness or death from COVID-19. According to recent recommendations from the Italian Ministry of Health and an Italian expert consensus, people with Multiple Sclerosis (MS), especially those with disabilities, progressive forms of the disease, older age, and comorbidities, were considered to be the category with the highest priority during the second phase of the Italian immunization programme (1, 2).

At this moment, very limited data on the effectiveness and safety profile of COVID-19 vaccines are available for MS patients. In this report, we describe a case of severe relapse occurred in a MS patient who had received COVID-19 vaccine. Written informed consent was obtained from the patient for the publication of any potentially identifiable images or data included in this article.

## Case Presentation

In November 2016, a 26-year-old woman presented to the MS Centre of the A. Cardarelli Hospital (Italy) with tinnitus and dizziness. The MRI showed multiple periventricular, brain stem, and spinal cord hyperintense T2/FLAIR lesions, some of which were active. She was diagnosed with MS and started the treatment with fingolimod. In June 2018, after experiencing a clinical relapse, she started the first cycle of cladribine, which was well-tolerated. At 6-month, the MRI showed two new periventricular T2/FLAIR lesions with enhancement. No new lesions were present in the 1-year follow-up brain MRI. In June 2019, the patient was treated with a second cycle of cladribine tablets. Until March 2021 she showed neither clinical nor radiological progression of disease.

**Figure 1:**
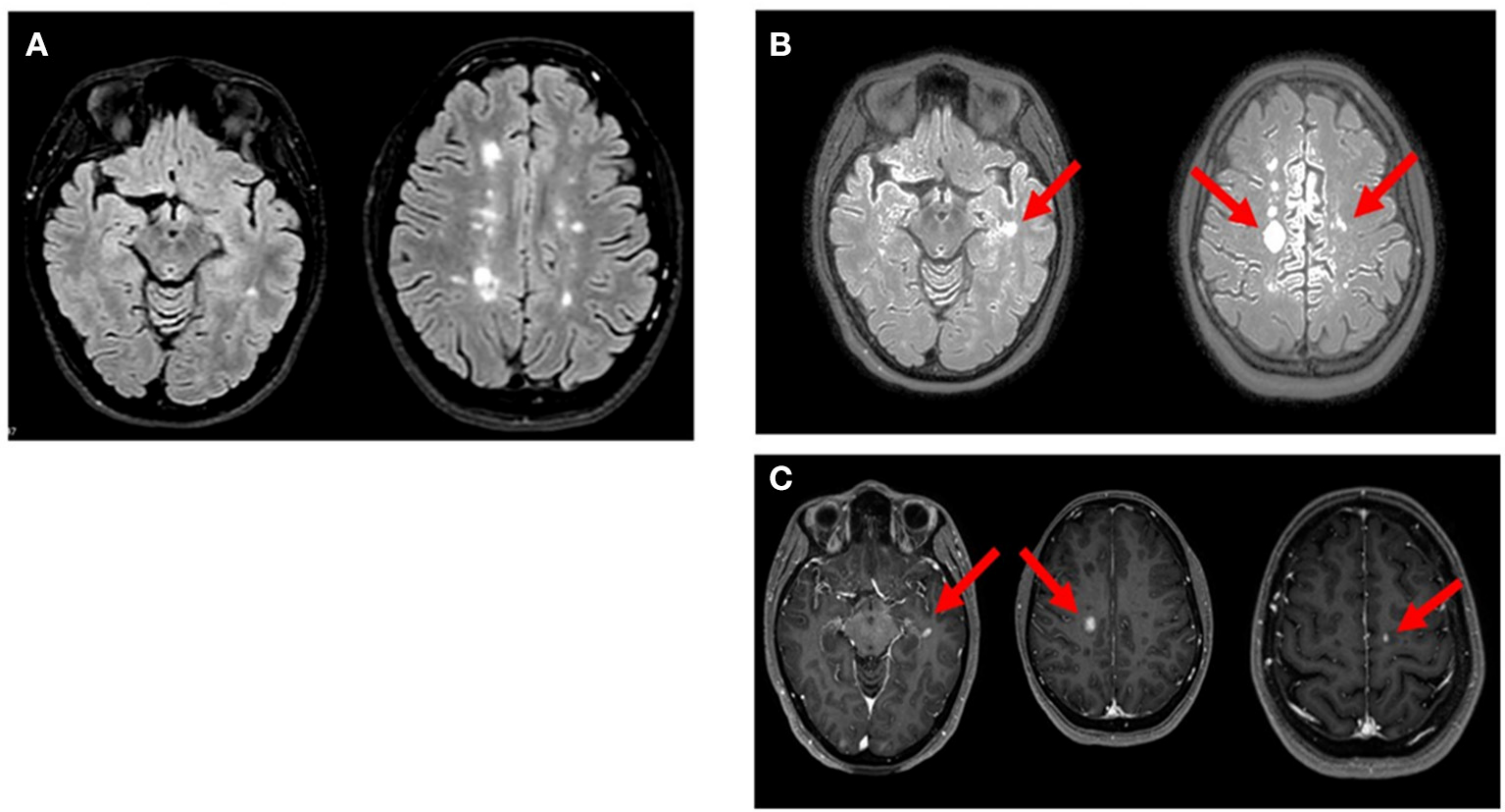
Axial MRI images of reported patient. (**A**; Oct 2020)**:** multiple hyperintense white matter lesions were observed in T2/FLAIR weighted scan. (**B**; Apr 2021)**:** T2/FLAIR weighted scan showed new three lesions in left temporal lobe and bilateral precentral cortex (arrows). (**C**; Apr 2021)**:** gadolinium-enhancing lesions (arrow) corresponding to the new hyperintense lesions on FLAIR were noted in T1 weighted postcontrast images.

On April 8th, 2021, she received COVID-19 BNT162b2 vaccine. Approximately 48 h after receiving the first dose of BNT162b2, the patient developed paraesthesia in her left arm followed by weakness in her left upper and lower limbs. Five days after the onset of symptoms, the neurological examination revealed walking difficulties and strength of 3/5 in the left upper limb and 2/5 in the left lower limb, left hyperreflexia in deep tendon reflexes and loss of vibrations in the left hemisome. The MRI showed three new voluminous enhancing lesions: left (11.5 × 8 × 8.5mm) and right (17 × 14 × 13mm) frontal cortical lesions and left temporal lesion (Figure 1). Blood tests before and after the vaccination were performed (Tables 1, 2). The patient received methylprednisolone iv for 5 days with full recovery of neurological symptoms. Due to the new relapse and the steroid treatment, we decided not to adhere to the vaccination schedule as the patient did not take the second dose of BNT162b2.

**Table 1:** Lymphocytes subsets before and after the vaccination and during the relapse.

	**Before the vaccine**	**During relapse**	**After 4 week the vaccine**
WBC	4.79 × 10^3^/ul	5.170 × 10^3^/ul	5.40 × 10^3^/ul
Lymphocytes	1.520 cell/ul	960 cell/ul	1.34 cell/ul
CD3	979.2 cell/ul	691.2 cell/ul	951.4 cell/ul
CD8	164.16 cell/ml	112.8 cell/ul	151.956 cell/ul
CD4	547.2 cell/ul	403.2 cell/ul	562.8 cell/ul
CD19	115.2 cell/ul	105.6 cell/ul	134 cell/ul
CD20	115.2 cell/ul	105.6 cell/ul	134 cell/ul
CD3/HLA-DR (activated lymphocytes)	60.8 cell/ul	19.2 cell/ul	53.6 cell/ul

**Table 2:** Ig anti-Spike SARS COV2 before and after the vaccination (range < 0.80).

	**Before the vaccine**	**After 4^**°**^ week the vaccine**
Ig against Spike SARS-COV-2	0.40 U/ml	17.14 U/ml

## Discussion

We described a case of severe acute relapse occurred in a young patient after mRNA-based COVID-19 vaccine. Among MS patients, relapses still represent one of the most unpredictable aspects to be managed. Indeed, their prompt recognition and treatment are essential in order to achieve good clinical outcomes. Many risk factors for acute relapse were identified, including female sex (women are more likely to experience relapses throughout the course of the disease), smoking status and the discontinuation of highly effective therapy (3–5).

Adverse events following mRNA-based COVID-19 vaccines are generally mild and consist in injection site reactions, headache and asthenia (6). However, data on the efficacy and safety of these vaccines in MS patients are rather limited (7). To our knowledge, only one observational study was carried out among 555 MS patients. The study was conducted in one clinical Centre in Israel where all patients received the BNT162b2 vaccine. The safety profile of COVID-19 vaccine resembled that observed in patients enrolled in premarketing clinical trials. Indeed, the most common AEFIs were injection site reactions, fatigue, and headache. Acute relapses were detected in 2.1% of patients after the first vaccine dose and in 1.6% of patients after the second dose. The comparison of these rates with those of previous years highlighted no differences, although the short follow-up period could have resulted in lower relapses' rate (8). In our case, the early onset of neurological symptoms after the vaccination questioned the causal association with the vaccine but the presence of multiple active lesions on brain MRI undoubtedly represents a recent activation of MS. In addition, mRNA-based COVID-19 vaccines might elicit a strong T and B cells response (9), which in turn could underpin the development of autoimmune processes (10). The immunophenotype performed during the relapse showed a mild lymphopenia, mainly affecting the activated T cell compartment, both CD4 and CD8 T cells (Table 1). This phenomenon could be secondary to increased migration and localization of autoreactive T cell into CNS during the relapse. After 4 weeks from vaccination antibodies against Spike-SARS-COV-2 protein were present, although at low titer (Table 2). As a pulse therapy, cladribine may not be able to contain a strong inflammatory response triggered by the vaccine, leading to a relapse. On the other hand, among DMTs, cladribine is associated with a low risk of infection and severe lymphopenia (11). In addition, since systemic infections, such as COVID-19, can worsen MS, the vaccination can be able to reduce the risk of relapses by dropping the risk of infections (8). Thus, at this moment, currently available data show that COVID-19 vaccines seem to be not associated with an increased risk of acute relapses.

To our knowledge this is the first case describing the occurrence of a severe acute relapse in a young patient after mRNA-based COVID-19 vaccine, even though cases of MS relapses occurred shortly after other COVID-19 vaccines can be found in the literature. Indeed, Etemadifar et al. described the case of a 34-year-old woman suffering from RRMS and treated with rituximab, who had received her first dose of adenovirus-vectored COVID-19 vaccine Gam-COVID-Vac (Sputnik V) 3 days before experiencing her latest MS relapse episode, preceded by mild symptoms (fatigue, myalgia, generalized weakness, etc.). As for our patient, the woman received corticosteroid therapy for five consecutive days, and her neurological deficits slightly improved. However, differently from the decision made by our neurologists, this woman received also her second dose of Gam-COVID-Vac after discontinuation of oral steroid taper (12). On the other hand, data from an interim analysis of the efficacy and safety of the ChAdOx1 nCoV-19 vaccine from four ongoing blinded, randomized, controlled trials (COV001, COV002, COV003, and COV005) reported a case of transverse myelitis occurred 10 days after the first dose of ChAdOx1 nCoV-19 in a patient with MS, that was determined to be unlikely to be related to vaccination by an independent committee of neurological experts (13). We are aware that further studies are necessary in order to better define the efficacy and safety profile of COVID-19 vaccines among MS patients, also in terms of risk of acute relapse.

## Conclusion

To our knowledge, this is the first report of acute relapse following COVID-19 vaccine in a patient with a long-standing history of MS. Current knowledge seems to suggest that MS patients had similar rates of AEFIs to the general population following BNT162b2 vaccination. Our case provides new evidence on COVID-19 vaccination in MS patients but new knowledge in this field is strongly needed. Thus, the strict monitoring of MS patients who are receiving now COVID-19 vaccination should be taken into account by neurologists during their routine clinical practice.

## Data Availability Statement

The original contributions presented in the study are included in the article/supplementary material, further inquiries can be directed to the corresponding author/s.

## Ethics Statement

Ethical review and approval was not required for the study on human participants in accordance with the local legislation and institutional requirements. The patients/participants provided their written informed consent to participate in this study. Written informed consent was obtained from the individual(s) for the publication of any potentially identifiable images or data included in this article.

## Author Contributions

GM, VM, MDB, SS, OM, CS, and AC: drafting the work and revising it for important intellectual content. GM, CS, and AC: substantial contributions to the acquisition, analysis, or interpretation of data for the work. GM, VM, MDB, SS, OM, CS, and AC: final approval of the version to be published. GM, VM, MDB, SS, OM, CS, and AC: agreement to be accountable for all aspects of the work in ensuring that questions related to the accuracy or integrity of any part of the work are appropriately investigated and resolved. GM and AC: developed the concept. GM, CS, and AC: wrote the paper. All authors contributed to the article and approved the submitted version.

## Conflict of Interest

GM received personal compensation from Serono, Biogen, Novartis, Roche, and TEVA for public speaking and advisory boards. The remaining authors declare that the research was conducted in the absence of any commercial or financial relationships that could be construed as a potential conflict of interest.

## Publisher's Note

All claims expressed in this article are solely those of the authors and do not necessarily represent those of their affiliated organizations, or those of the publisher, the editors and the reviewers. Any product that may be evaluated in this article, or claim that may be made by its manufacturer, is not guaranteed or endorsed by the publisher.

## References

[1] Ministero della salute: Raccomandazioni ad interim sui gruppi target della vaccinazione anti-SARS-CoV-2/COVID-19 8 Febbraio 2021. Available online at: https://www.trovanorme.salute.gov.it/norme/renderPdf.spring?seriegu=SG&datagu=24/03/2021&redaz=21A01802&artp=1&art=1&subart=1&subart1=10&vers=1&prog=002 (accessed July 26, 2021).

[2] Centonze D Rocca MA Gasperini C Kappos L Hartung H-P Magyari M. Disease-modifying therapies and SARS-CoV-2 vaccination in multiple sclerosis: an expert consensus. J Neurol. (2021) 1-8. 10.1007/s00415-021-10545-2. [Epub ahead of print]. 33844056 PMC8038920

[3] Maniscalco GT Brescia Morra V Florio C Lus G Tedeschi G Cianfrani M. Preliminary results of the FASM study, an on-going Italian active pharmacovigilance project. Pharmaceuticals (Basel). (2020) 13:466. 10.3390/ph13120466 33333889 PMC7765255

[4] Maniscalco GT Saccà F Lanzillo R Annovazzi P Baroncini D Binello E. First therapy choice in newly diagnosed multiple sclerosis patients: a multicenter Italian study. Mult Scler Relat Disord. (2020) 42:102059. 10.1016/j.msard.2020.102059 32208344

[5] Tremlett H Zhao Y Joseph J Devonshire V. UBCMS clinic neurologists: relapses in multiple sclerosis are age- and time-dependent. J Neurol Neurosurg Psychiatry. (2008) 79:1368-74. 10.1136/jnnp.2008.145805 18535026

[6] CDC. Possible Side Effects After Getting a COVID-19 Vaccine. Available online at: https://www.cdc.gov/coronavirus/2019-ncov/vaccines/expect/after.html (accessed June 15, 2021).

[7] Chilimuri S Mantri N Gongati S Zahid M Sun H. COVID-19 vaccine failure in a patient with multiple sclerosis on ocrelizumab. Vaccines (Basel). (2021) 9:219. 10.3390/vaccines9030219 33806646 PMC8002140

[8] Achiron A Dolev M Menascu S Zohar DN Dreyer-Alster S Miron S. COVID-19 vaccination in patients with multiple sclerosis: what we have learnt by February 2021. Mult Scler. (2021) 15:13524585211003476. 10.1177/13524585211003476 33856242 PMC8114441

[9] Sahin U Muik A Derhovanessian E Vogler I Kranz LM Vormehr M. COVID-19 vaccine BNT162b1 elicits human antibody and TH1 T cell responses. Nature. (2020) 586:594-9. 10.1038/s41586-020-2814-7 32998157

[10] Petersone L Edner NM Ovcinnikovs V Heuts F Ross EM Ntavli E. T cell/B cell collaboration and autoimmunity: an intimate relationship. Front Immunol. (2018) 9:1941. 10.3389/fimmu.2018.01941 30210496 PMC6119692

[11] Maniscalco GT Annunziata M Ranieri A Alfieri G Renna R Iorio WD. Remission of early persistent cladribine-induced neutropenia after filgrastim therapy in a patient with relapsing - remitting multiple sclerosis. Mult Scler Relat Disord. (2020) 43:102151. 10.1016/j.msard.2020.102151 32417665

[12] Etemadifar M Sigari AA Sedaghat N Salari M Nouri H. Acute relapse and poor immunization following COVID-19 vaccination in a rituximab-treated multiple sclerosis patient. Hum Vaccin Immunother. (2021) 20:1-3. 10.1080/21645515.2021.1928463 34015240 PMC8437516

[13] Voysey M Clemens SAC Madhi SA Weckx LY Folegatti PM Aley PK. Safety and efficacy of the ChAdOx1 nCoV-19 vaccine (AZD1222) against SARS-CoV-2: an interim analysis of four randomised controlled trials in Brazil, South Africa, and the UK. Lancet. (2021) 397:99-111. 10.1016/S0140-6736(20)32661-1 33306989 PMC7723445

